# Regulation of Translation, Translocation, and Degradation of Proteins at the Membrane of the Endoplasmic Reticulum

**DOI:** 10.3390/ijms23105576

**Published:** 2022-05-17

**Authors:** Lea Daverkausen-Fischer, Margarethe Draga, Felicitas Pröls

**Affiliations:** Institute of Anatomy II, Medical Faculty and University Hospital Cologne, University of Cologne, Joseph-Stelzmann Str. 9, D-50931 Cologne, Germany; leavaldf@gmail.com (L.D.-F.); margarethe.draga@uk-koeln.de (M.D.)

**Keywords:** UPR, ERAD, co-chaperones, translocation, degradation, translocon

## Abstract

The endoplasmic reticulum (ER) of mammalian cells is the central organelle for the maturation and folding of transmembrane proteins and for proteins destined to be secreted into the extracellular space. The proper folding of target proteins is achieved and supervised by a complex endogenous chaperone machinery. BiP, a member of the Hsp70 protein family, is the central chaperone in the ER. The chaperoning activity of BiP is assisted by ER-resident DnaJ (ERdj) proteins due to their ability to stimulate the low, intrinsic ATPase activity of BiP. Besides their co-chaperoning activity, ERdj proteins also regulate and tightly control the translation, translocation, and degradation of proteins. Disturbances in the luminal homeostasis result in the accumulation of unfolded proteins, thereby eliciting a stress response, the so-called unfolded protein response (UPR). Accumulated proteins are either deleterious due to the functional loss of the respective protein and/or due to their deposition as intra- or extracellular protein aggregates. A variety of metabolic diseases are known to date, which are associated with the dysfunction of components of the chaperone machinery. In this review, we will delineate the impact of ERdj proteins in controlling protein synthesis and translocation under physiological and under stress conditions. A second aspect of this review is dedicated to the role of ERdj proteins in the ER-associated degradation pathway, by which unfolded or misfolded proteins are discharged from the ER. We will refer to some of the most prominent diseases known to be based on the dysfunction of ERdj proteins.

## 1. Introduction

Environmental changes, such as temperature shifts, nutritional deficits, or changes in redox conditions, cause imbalances in a cell’s ionic and protein homeostasis. Within the endoplasmic reticulum (ER), this leads to the aggregation of misfolded proteins, thereby eliciting ER stress. ER stress is sensed by a sophisticated system of transmembrane proteins, the inositol requiring enzyme 1α (IRE1α), protein kinase RNA-like endoplasmic reticulum kinase (PERK), and activating transcription factor 6 (ATF6), which are silenced by their binding to immunoglobin binding protein (BiP), which is the ER-resident heat shock 70 protein (Hsp70) protein. Vice versa, the activation of IRE1α, PERK, and ATF6 is induced by the release of BiP from their luminal domains and even by the direct binding of misfolded proteins to the luminal domains, as has been shown for IRE1α [1,2,3,4]. The activation of these proteins induces different pathways of a cell’s stress response, termed the unfolded protein response (UPR). UPR pathways aim to upregulate the levels of chaperones and foldases in the ER to increase the luminal folding capacity. In addition, the luminal protein burden is lowered by a general halt of protein synthesis, which is achieved by phosphorylation of the alpha 1 unit of the eukaryotic initiation factor (eIF2α) and mediated by the PERK arm of the UPR [5]. Furthermore, the IRE1α pathway promotes the degradation of luminal proteins by upregulating components of the ER-associated degradation (ERAD) pathway [6]. In order to clear the translocon from proteins whose translocation is impaired or delayed at the onset of stress, proteins are re-extracted from the translocon by a mechanism termed cotranslocational degradation [7,8]. All these processes are controlled and assisted by the endogenous chaperone machinery.

In mammalian cells, the import of proteins into the ER occurs via the Sec61 translocon. Active translocons are ribosome-associated and have a wide central pore of 4–6 nm in diameter [9], whereas inactive, ribosome-free translocons have a narrow pore of 0.9–1.5 nm in diameter [9]. The ribosome-induced conformational change, the tight association of the ribosome with the ER membrane, and a series of hydrophobic residues in the center of the pore help to form a seal during protein translocation. This seal limits the diffusion of small molecules into the cytosol, especially of the volatile Ca^2+^, which serves as a second messenger and controls a large variety of metabolic pathways [10,11]. BiP is also involved in gating the translocon. In its ADP-bound form, BiP is involved in closing the translocon while the opening of the gate is mediated by ATP-bound BiP [9,12,13,14]. Nucleotide binding to BiP is a central and typical theme of all Hsp70 proteins [15]. As a member of the Hsp70 protein family, the ER resident BiP consists of an N-terminal substrate-binding domain (SBD) and a C-terminal nucleotide-binding domain (NBD). The SBD binds unfolded or misfolded proteins. The binding of ATP to the NBD of BiP induces an allosteric conformational change at the SBD, which results in a low substrate affinity, whereas the binding of ADP to the NBD induces a high affinity to substrates. The transition of ADP-BiP to ATP-BiP is mediated by the nucleotide exchange factor Bap (Sil1 in yeast) [16,17]. By fluorescent quenching assays using isolated, reconstituted microsomes, the gating activity of BiP was analyzed. Interestingly, translocon closure is achieved by ADP-BiP, in which the SBD does not bind to an unfolded protein but, presumably, to one of the translocon components [13]. It was further shown that, for pore closure, ADP-BiP must interact with a membrane-bound J-domain protein at or near the translocon pore [9,13]. This is very interesting, since the J-domain is conventionally known to be required for stimulating the ATPase activity of ATP-BiP during the protein folding process. Considering these mechanisms, an ATPase-independent and an ATP-dependent function of BiP becomes evident. The ATPase-independent function of BiP (in its ADP-bound conformation) is required to close the gate, thereby limiting protein import into the ER. This “silencing” function of BiP is well-known for the UPR-mediating transmembrane proteins PERK, IRE1α, and ATF6, which are silenced by the binding of BiP to their respective luminal domains [1,3,13,18]. The ATPase-dependent activity of BiP encompasses the opening of the gate, which requires ATP-BiP as a driving force for translocation [13]. At the gate, BiP seems to act as a “molecular ratchet” to facilitate the transport of polypeptide chains across the membrane [19,20].

The BiP-ATP cycle described above is also required for the efficient folding of proteins. This chaperoning activity is ATPase-dependent. The low intrinsic ATPase activity of BiP can be largely stimulated by co-chaperones, which are also known as Hsp40 proteins, J-domain proteins (due to the highly conserved J-domain), or ERdj proteins for those co-chaperones, which are specifically located in the ER. Eight members of ERdj proteins, termed ERdj1–ERdj8, have been identified so far.

Besides their co-chaperoning activity, ERdj proteins are known to exert a variety of other functions. In this review, we will delineate the involvement and function of the ERdj proteins with respect to their role in controlling protein synthesis and the translocation of proteins into the ER. A second aspect of this review is dedicated to the role of the ERdj proteins in the ERAD pathway, by which unfolded or misfolded proteins are discharged from the ER. A selection of metabolic diseases is listed, which are based on the dysfunction of ERdj proteins.

## 2. Control of Protein Synthesis by ERdj1, ERdj2, and ERdj6

Three ERdj co-chaperones, ERdj1, ERdj2, and ERdj6, are functionally involved in the control of translation and translocation of ER target proteins [7,21,22,23,24,25]. In the following section, we will first focus on the control of protein synthesis and, then, on the regulation of protein translocation through the Sec61 translocon.

### 2.1. Translational Arrest Is Regulated by ERdj1 and ERdj2

**Figure 1:**
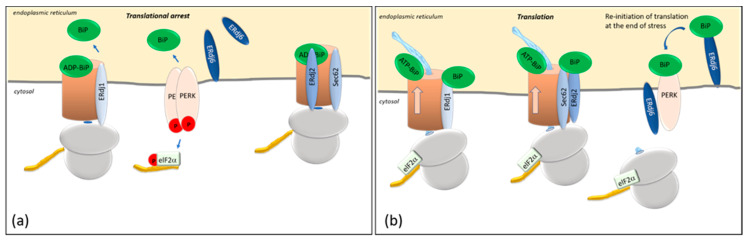
(**a**) At the translocon (brown cylinder), the pore is gated by BiP, which, in its ADP-bound form, closes the gate and opens it in its ATP-bound form. Translational arrest occurs when BiP dissociates from the luminal domains of ERdj1, ERdj2, and PERK. The release of BiP from PERK results in the dimerization of PERK, its autophosphorylation, and the subsequent phosphorylation of eIF2α (p-eIF2α), which inhibits eIF2α-dependent translation. (**b**) The translation of proteins is controlled by the co-chaperones ERdj1, ERdj2/Sec62, and ERdj6. In their BiP-bound forms, ERdj1 and ERdj2/Sec62 enable protein synthesis. The silencing of PERK and the PERK-signaling pathway occur by the binding of BiP to its luminal domain, which results in the release of eIF2α-dependent translation and protein synthesis. BiP, immunoglobin binding protein; PERK, protein kinase RNA-like endoplasmic reticulum kinase; eIF2α, alpha subunit of eukaryotic initiation factor 2.

ERdj1 is a transmembrane protein with its N-terminus in the ER lumen and its C-terminus in the cytosol [21,26]. ERdj1 inhibits protein synthesis at the Sec61 translocon in a BiP- and ATP-dependent way (Figure 1a). In vitro translation assays using preprolactin and luciferase as substrates suggested that the inhibition of protein synthesis is achieved by the first 21 N-terminal amino acids of the cytosolic domain of ERdj1, which bind to ribosomes close to the tunnel exit and to proteins emerging at the tunnel exit [21,22,23,24]. Translation is arrested at its initiation, since ERdj1 is not able to inhibit the translation of preprolactin when added after translational initiation [23]. The inhibitory function of ERdj1, as well as ribosome binding, seems to be mediated by a four-amino acid RKKR within the RKKRERKKK stretch [23]. This polybasic amino acid stretch is similar to an amino acid stretch within the SRP, which also inhibits elongation [22,27].

In the presence of ERdj1, an increased amount of BiP associates with the ribosomal fractions, suggesting that ERdj1 plays a role in recruiting BiP to the ribosome/ERdj1 complex [21,23]. A BiP mutant (BiP R197H), which is unable to bind to J-domains and experiments with the J-domain-deleted ERdj1 protein, revealed that the recruitment of BiP to the ERdj1–ribosome complex is ATP-dependent and requires the functional interaction of BiP and the ATPase-stimulating J-domain [23]. The association of ERdj1 with the emerging protein and, accordingly, the translational arrest was shown to occur only in the absence of BiP [23] (Figure 1a). Upon the binding of BiP to ERdj1, the inhibition of protein translation by ERdj1 is released [21,23] (Figure 1b). ERdj1 thus seems to relay the information about luminal BiP availability to the translating ribosomes at the ER membrane. It is unclear whether the binding of BiP and ribosomes to ERdj1 occur simultaneously or sequentially. Dudek et al. showed that ERdj1 can simultaneously interact with the ribosome and BiP and that the binding of BiP promotes the re-initiation of translation [23]. On the other hand, experimental data from surface plasmon resonance show that the binding of BiP to ERdj1 prevents the binding of ERdj1 to ribosomes [24], indicating that the ERdj1/ribosome complex needs to be established before BiP can bind to it. In any case, BiP abolishes the inhibitory effect of ERdj1 on protein translation, whether or not the binding of BiP to ERdj1 occurs simultaneously or sequentially [23,24]. It is also still unclear how BiP binding to ERdj1 is signaled to the cytosol to release the translational arrest [22]. Additionally, the data from surface plasmon resonance, which demonstrated that the prebinding of BiP to ERdj1 inhibits ribosome binding to ERdj1, are difficult to understand, given the fact that ERdj1 has a much higher affinity for ribosomes (K_D_ 30 pM) than for BiP (K_D_ 0.57µM) [21,28]. A possible mechanism could be that the prebinding of BiP to ERdj1 alters the affinity of ERdj1 for ribosomes. The fact that ERdj1 can inhibit the translation until BiP binds to ERdj1 could present a mechanism by which the ER luminal balance of the chaperone and protein levels is ensured. When a large number of unfolded proteins prevails within the ER, BiP is released from ERdj1 to bind to client proteins, thereby inducing an ERdj1-mediated translational arrest (Figure 1a). When client proteins are folded successfully, BiP is again available to bind to ERdj1 so that the translation can progress [23] (Figure 1b). Translational arrest is a known mechanism of reducing ER stress during the UPR, mainly mediated by the ER stress sensor PERK [29] (Figure 1). The PERK-dependent phosphorylation of eIF2α inhibits the formation of the translational initiation complex [29,30]. This results in the inhibition of general, eIF2α-dependent protein translations, so that the protein load in the ER is reduced [29]. Given this analogy, ERdj1 presumably acts as an additional sensor of ER stress [23]. The dissociation constant of the ERdj1/BiP complex in the presence of ATP is 0.57 µM, whereas the dissociation constant of the PERK luminal domain/BiP complex is 0.83 µM [24,31], which allows a fine tuning of translational arrest to the amount of accumulating, BiP-consuming unfolded proteins. According to these results, BiP would dissociate more easily from PERK than from ERdj1, and ERdj1-mediated translational inhibition would sequentially follow PERK-mediated translational inhibition (Figure 1b). Yet, it has to be noted that the experimental conditions do not really reflect the in vivo situation. Neither of the experiments were performed with PERK or ERdj1 in their original topology as integral membrane proteins [24,31]. Additionally, different methods were used to determine the affinity of BiP to ERdj1 or to PERK using surface plasmon resonance [24] or microscale thermophoresis [31], respectively.

In conclusion, determination of the dissociation constants of PERK/BiP and ERdj1/BiP in their original topologies with identical experimental methods should be performed, since these values are critically important for a reliable evaluation of the discussed sequential BiP release from its stress sensors ERdj1 and PERK.

The co-chaperone ERdj2, in conjunction with Sec62, also controls protein translation (Figure 1). Like ERdj1, Sec62 associates with emerging proteins close to the tunnel exit of the 60S ribosomal subunit [22,28]. Due to its association with the translocon-associated protein Sec62, ERdj2 is also termed Sec63 [25,28,32]. The interaction of Sec62 and ERdj2 has been investigated by pulldown assays and surface plasmon resonance spectroscopy. Accordingly, the C-terminal residues (aa734–aa760) of ERdj2 interact with the N-terminal residues (aa11–aa155) of human Sec62. The weak binding of both proteins was shown to be strengthened by CK2-mediated phosphorylation [25,28,32,33]. Pulldown experiments revealed that the binding of Sec62 to ribosomes weakens the binding of Sec62 to ERdj2 and that Sec62 is displaced from ERdj2 upon binding to ribosomes [28]. This is in accordance with the data showing a higher affinity of Sec62 to ribosomes (K_D_ 0.13 nM) than to ERdj2 (K_D_ 5 nM) [28,34]. Furthermore, the ribosome-binding site is close to the N-terminal, ERdj2-binding region of Sec62, as was shown in in vitro ribosome binding assays and fractionation experiments [28]. Interestingly, co-immunoprecipitation experiments using microsomal extracts could precipitate Sec62 due to its binding to ERdj2 but not vice versa [25,32]. The reason for this is unknown. Yet, the experiments in which the co-immunoprecipitation of ERdj2 with Sec62 failed were performed with antibodies, which were directed against the C-terminus of Sec62 [25,32]. It can be assumed that the binding of the precipitating antibody induces a conformational change at the N-terminus of Sec62, thereby disabling the binding of Sec62 to ERdj2 [28].

Taken together, two functional pools of Sec62 seem to exist at the ER membrane, with Sec62 being either associated with or displaced from ERdj2. When displaced from ERdj2, Sec62 is bound to ribosomes, a conformation in which protein synthesis and translocation is inhibited.

Interestingly, a functional redundancy exists between ERdj1 and Sec62 in that both proteins bind to the same region at the ribosomal tunnel exit. The preincubation of ribosomes with the C-terminus of ERdj1 (ERdj1C) prevents binding of the N-terminus of Sec62 (Sec62N) to ribosomes [28]. The binding of Sec62N to a translating ribosome can inhibit the initiation of protein translation in a strikingly similar manner as the binding of ERdj1 to translating ribosomes. The interaction of Sec62N with ribosomes is, as the interaction of ERdj1 with ribosomes, RNase- and salt-sensitive and can be abolished at a KCl concentration of 300 mM [24]. Due to the functional similarity between ERdj1 and the Sec62N–ERdj2 complex, overlapping functions at the ER membrane might explain the fact that the loss of ERdj2 function does not cause a lethal phenotype [24]. In this context, it would be interesting to examine the effect of ERdj1/Sec62 or ERdj1/ERdj2 double knockouts. Furthermore, it would be interesting to know how ERdj2 binding to Sec62 affects Sec62 binding to ribosomes and how BiP binding to ERdj2 affects the ERdj2/Sec62/ribosome complex.

### 2.2. ERdj6 Reinitiates Translation at the End of a Stress Period

**Figure 2:**
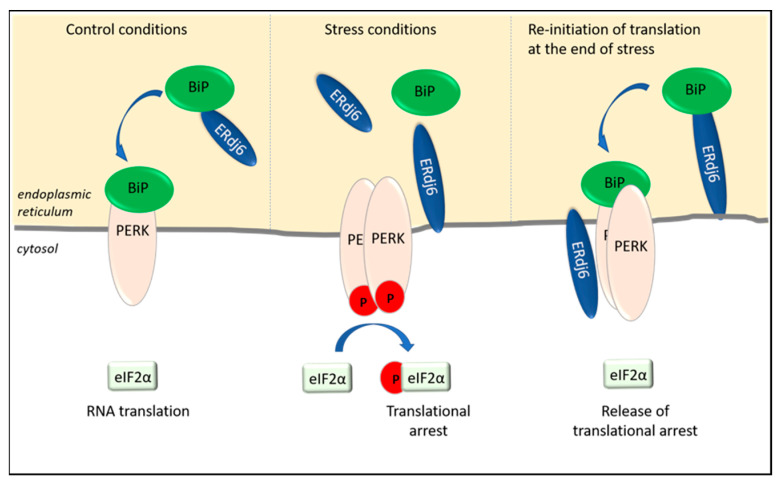
ERdj6 surveys the activation state of PERK. ERdj6 can adopt various subcellular localizations. Under control conditions, ERdj6 is located luminally and transfers BiP to PERK to keep PERK in a silenced state and to enable eIF2α-dependent RNA translation. In ER-stressed cells (as observed after 8 h thapsigargin treatment), ERdj6 is inserted in the ER membrane. Half of the membrane-anchored pool faces the lumen, and half of the pool faces the cytosol. Due to the increased demand of chaperoning activity, BiP dissociates from the luminal domain of PERK and enables the activation and autophosphorylation of PERK. The subsequent phosphorylation of eIF2α inhibits eIF2α-dependent translational processes. The translational arrest can be released by interaction of the cytosolic ERdj6 pool with the C-terminus of PERK (see text).

Apart from ERdj1 and ERdj2, additional translational control is exerted by the co-chaperone ERdj6, which is upregulated in ER-stressed cells [35,36] (Figure 1). The upregulation of ERdj6 was reported to inhibit the amount of phosphorylated PERK, as well as phosphorylated eIF2α, in cells, thus alleviating the inhibitory effect of PERK on RNA translation and protein synthesis [35]. The stimulation of protein synthesis has been shown in HeLa cells, as well as in HEK293 cells, in which the downregulation of ERdj6 results in the downregulation of general protein synthesis [36,37]. This is interesting, since *ERdj6* transcription and ERdj6 synthesis themselves are upregulated in stressed cells [35,36]. Accordingly, in stressed cells, the upregulated ERdj6 levels silence PERK, thereby releasing PERK-mediated translational arrest [35] and re-adjusting the cellular metabolism towards increased rates of protein synthesis (Figure 2). This scenario must be considered as a feedback mechanism to normalize cellular physiology at the end of a stress period. This hypothesis is supported by data indicating a switch in the topology of ERdj6 upon ER stress. In cell lysates of control cells transfected with ERdj6, which was engineered to contain a C-terminal glycosylation site (-CHO mutant), EndoH treatment, as well as a proteinase K experiment, revealed that the entire ERdj6-CHO protein pool—with a cleaved signal sequence—seems to be located in the ER lumen [37,38]. After 8 h of thapsigargin-mediated ER stress, a membrane-anchored ER luminal pool appears beside the free luminal pool, and after 24 h of thapsigargin treatment, the only protein pool present contains the uncleaved membrane signal, with one-half being glycosylated and the other half unglycosylated and susceptible to proteinase K treatment [37]. So, while under control conditions, ERdj6 is mainly present as a luminal ER resident protein, the treatment with thapsigargin increases the pool of ER signal peptide-uncleaved ERdj6 protein, located luminally as well as cytosolically [26,37]. Since ERdj6 co-immunoprecipitates with full-length PERK but not with a construct lacking the cytosolic domain of PERK, it was suggested that ERdj6 exerts its inhibitory function on PERK by directly binding to the cytosolic kinase domain of PERK [35] (Figure 2).

On the other hand, the luminal ERdj6 pool also has the potential to silence PERK by promoting the formation of the BiP/PERK complex. According to the estimated dissociation constants, BiP has a greater affinity to PERK (K_D_ 0.83 µM) than to ERdj6 (K_D_ 5 µM) [31,39]. Hence, as schematically shown in Figure 2, ERdj6 could recruit and transfer BiP to PERK similar to the models suggested for ERdj2 (see above), as well as for ERdj4, which was shown to act as the BiP donor for IRE1, thereby keeping the respective signaling pathways in a silenced state [40,41]. Still, no experimental evidence has been presented so far that would support such a model.

All in all, the Hsp40 co-chaperones ERdj1 and ERdj2 (as the ERdj2/Sec62 complex) arrest protein translation in the absence of available BiP by direct association with the ribosome, while elevated ERdj6 levels in stressed cells promote protein translation by alleviating PERK-mediated translational arrest.

## 3. Co- and Posttranslational Translocation in Mammalian Cells by ERdj2 and ERdj6

Protein import into the ER occurs either co- or post-translationally via the Sec61 translocon. The majority of proteins is translocated cotranslationally, and only around 200 mammalian proteins are estimated to be translocated in a posttranslational mode [42]. Cotranslational transport is reported for proteins that contain an N-terminal hydrophobic stretch. As soon as about 70 amino acids are synthesized, the hydrophobic stretch emerges from the ribosome, is recognized by the signal recognition particle (SRP), and targeted—together with its bound mRNA and the nascent signal peptide—to the ER membrane, where the protein is further cotranslationally translocated [42]. Posttranslational translocation is mainly used by small presecretory proteins (smaller than 70 amino acids) and tail-anchored (TA) proteins, which—as described in mammals—contain their hydrophobic stretch at the C-terminus [42]. As soon as the hydrophobic stretch emerges from the ribosome, these proteins are also targeted by the SRP and transported to the translocon for their translocation [42,43]. Slight variations in this general rule are reported for prolactin (PRL) and the prion protein (PrP). The cotranslational translocation of PRL does not initiate prior to the translation of about 163 amino acids, which are translated in the cytosol [44]. The import of PrP, which is also translocated cotranslationally, initiates only after translation of the first 188–207 amino acids [44]. To prevent aggregation of the proteins in the cytosol, cytosolic Hsc70 proteins and J-proteins bind to and shield the aggregation-prone sites of the newly synthesized proteins [42]. The translocation of proteins across the ER membrane is assisted by co-chaperones, either in an ERdj2-dependent or -independent manner. In the following section, we will decipher the role of ERdj2 in controlling co- and posttranslational translocation.

### 3.1. Features Determining ERdj2-Mediated Cotranslational Translocation of Proteins

The posttranslational translocation of secretory proteins is promoted by ERdj2 and Sec62, as has been well-established in yeast [42]. Yet, the downregulation of ERdj2 in mammalian cells only slightly affected the posttranslational translocation of the small secretory protein PcecA [45]. Even though a transient association of ERdj2 with tail-anchored (TA) proteins was observed during translocation, the downregulation of ERdj2 did not impair the posttranslational translocation of a set of TA proteins [45,46,47,48]. Overall, a larger number of post-translationally translocated proteins must be examined before a general statement on the role of ERdj2 in posttranslational translocation in mammalian cells can be made.

In mammalian cells, the majority of proteins are translocated across the Sec61 translocon in a cotranslational way. The impact of ERdj2 and Sec62 on cotranslational translocation has been shown for a variety of proteins, such as the invariant chain of the human class II major histocompatibility complex, aquaporin 2, two derivates of PrP, and even the translocation of the co-chaperone ERdj3 [45]. Since the cytosolic domain of ERdj2 was shown to clash with the large ribosomal subunit, it is suggested that proteins, dependent on ERdj2 for translocation, partially fold in the cytosol, thereby mediating a partial release of the ribosome from the translocon to enable the binding of ERdj2 [49]. Since ERdj2 dependency during cotranslational translocation was not observed for all the cotranslationally translocated proteins examined [45], the underlying mechanisms for cotranslational control become increasingly interesting. It is assumed that ERdj2 dependency is ruled by the concerted interaction of the signal sequence [14,45,50] and the length of the protein [46] in conjunction with the structure of an internal region within the mature protein [14,50,51]. We will refer to these parameters in the following sections.

For the investigation of the impact of the length of the target protein on ERdj2 dependency, the small ERdj2-dependent proteins preproapelin and prestatherin were modified by the introduction of a DHFR domain that extended the protein length. This modification partially allowed for ERdj2-independent cotranslational translocation into the ER, arguing for a role of the protein length in ERdj2 dependency [51]. With regards to PrP, truncated constructs of the protein were equally dependent on ERdj2 as the full-length wildtype protein [14], indicating that protein length alone is not a unique factor critical for ERdj2-dependent translocation.

Investigation of the signal sequence on ERdj2-dependent translocation efficiency showed that the nature and strength of signal sequences is another factor that determines ERdj2 dependency. While the exchange of the ERdj2-dependent signal peptide of PcecA (ERdj2-dependent translocation) by the signal sequence of PpαF (ERdj2-independent translocation) did not alter the ERdj2 dependency of PcecA translocation [46], the ERdj2-dependent translocation of PrP became ERdj2-independent after the exchange of its signal sequence with the signal sequence of prolactin (PRL), the translocation of which is ERdj2-independent [14,52]. The substitution of the signal sequence of the small preproapelin (ERdj2-dependent protein) for that of PRL (ERdj2-independent protein) conferred ERdj2 independency to preproapelin in HeLa cells [51]. Similar results were observed when the ERdj3 signal sequence was exchanged for that of PRL [50]. Taken together, these results confirm that signal sequences not only target proteins to the ER, but they also determine translocation efficiencies. Strong signal sequences like the one of PRL confer ERdj2 independency to a protein and target the entire protein pool to the ER.

A major factor determining ERdj2-dependent translocation seems to be the nature of the region positioned next to the signal peptide. Positive charges next to the signal peptide seem to require the assistance of ERdj2 for cotranslational translocation. The deletion of positively charged amino acids adjacent to the signal peptide resulted in the ERdj2-independent translocation of a truncated PrP [14] and, correspondingly, to an ERdj2-independent translocation of a mutated preproapelin devoid of its basic amino acid-rich domain [51].

Steric hindrance is also reported to affect cotranslational translocation and to determine the fate of proteins at the translocon. The translocation of preprolactin, which contains a strong signal sequence and is independent of ERdj2, is arrested at the translocon after the insertion of a zinc finger domain into the preprolactin sequence [44]. Although ERdj2 is stably associated with this zinc finger domain-containing preprolactin [44], it does not succeed in moving this mutant across the membrane. It would be of interest to know whether the binding of ERdj2 occurs due to the delay of the zinc finger-containing preprolactin at the translocon or whether it binds to preprolactin due to the positive charges added by the insertion of the zinc finger domain. It must be assumed that proteins that are stuck in the translocon are targeted to proteasomal degradation [8,53]. This cotranslational degradation is largely mediated by ERdj6, as is outlined in the next section.

### 3.2. ERdj6-Mediated Cotranslational Degradation

**Figure 3:**
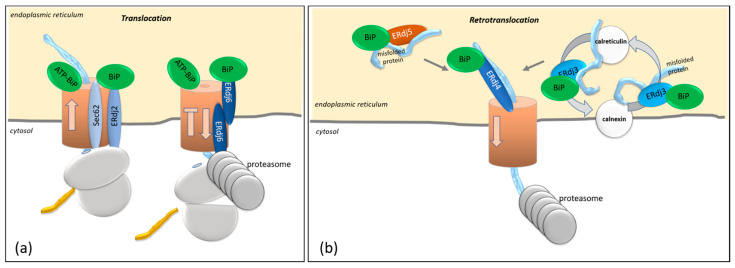
(**a**) Cotranslational translocation of proteins occurs via the Sec61 translocon and is mediated in a BiP-dependent way either in the absence or presence of ERdj2, depending on the respective target protein. Cotranslational degradation of a target proteins is controlled by ERdj6. The retrotranslocation of targets that clog the translocon is BiP-dependent. (**b**) The degradation of luminal target proteins is mediated by ERdj3, ERdj4, and ERdj5. ERdj3 seems to compete with target proteins for the binding of calnexin or calreticulin and controls the degradation or folding of proteins. ERdj4 also mediates either the folding of proteins or, in the case of enduring misfolding, targets the protein to the ERAD pathway. The specific feature of ERdj5 is its reductive ability, required to release disulifide bonds in order to enable a subsequent retrotranslocation across the ER membrane.

After chemical inhibition of translocation, proteins at the translocon were shown to be degraded. This process is termed cotranslational degradation and clears the translocon in stressed cells. Experimental data revealed a decisive role of the co-chaperone ERdj6, which is associated with the translocon [7], in the process of cotranslational degradation. As outlined below, the overexpression of ERdj6 promoted the cotranslational degradation of several proteins [7,53] (Figure 3a). ERdj6 limits the protein translocation of TCRα, as indicated by experiments in which higher levels of the ER resident glycosylated TCRα were obtained after the downregulation of ERdj6, even though no accelerated protein degradation occurred [7]. Additionally, elevated levels of the ERdj3 protein were observed in response to ERdj6 downregulation [39]. Vice versa, the overexpression of ERdj6 resulted in the impaired translocation of PrP and various PrP constructs [7,8,37], implying that ERdj6 restricts protein translocation into the ER. The J-domain, as well as the signal peptide of ERdj6, were shown to be necessary for ERdj6 to fulfil its function as a regulator of protein translocation [7,37]. The question of how ERdj6 exerts its function as a regulator of protein translocation has been addressed in the past and is still under debate. In vitro experiments using purified GST-ERdj6 showed that ERdj6 forms a complex with the cytosolic Hsp70 and that ERdj6/Hsp70 complexing is enhanced in the presence of ATP [7]. Consistent with these data, Oyadomari et al. showed that ERdj6 coelutes with Hsp70 in the presence of ATP when detergent-solubilized ER membranes are loaded onto an ATP–agarose affinity matrix. Co-elution was also observed in digitonin-solubilized ER membrane preparations, in which the integrity of the translocon was preserved [7]. Accordingly, ERdj6 seems to recruit Hsp70 to the cytosolic side of the translocon [7]. These data argue for the cytosolic ERdj6 pool being responsible for translocational control [7]. Yet, a chimeric construct with the efficiently translocated prolactin signal peptide (PRL-ERdj6) was shown to limit the translocation of PrP, huCD4, PTK7, or the luminal protein ERLEC1/XTP3B, arguing for the luminal ERdj6 pool to control substrate translocation [37]. These data have been confirmed recently by Pauwels et al., showing that the luminal pool of ERdj6 destabilizes targets that are stuck in the translocon during the process of cotranslational translocation. This effect is most prominent with the soluble luminal target ERLEC1/XTP3B and less pronounced with integral membrane proteins [53]. It can be assumed that luminal ERdj6 titrates BiP away from other co-chaperones like ERdj2, which are required for the translocation of specific target proteins [37,45]. This hypothesis is supported by data showing that ERdj6 is upregulated after six hours of ER stress induced by tunicamycin treatment while ERdj2 is not [6,54]. Furthermore, it seems that the targets, which depend on ERdj2-mediated translocation, are the ones that are recognized and degraded by ERdj6 in the case of failed translocation. When acting on identical target proteins but exerting opposing functions, ERdj2 and ERdj6 might constitute a kind of yin/yang partnership [8,14].

As the luminal ERdj6 was also shown to promote the maturation and folding of substrate proteins [37,38], it is rather likely that the ER luminal ERdj6 pool exerts three functions (i) as a co-chaperone for BiP to ensure proper protein folding, (ii) to control protein translocation in cooperation with ERdj2, and (iii) as a regulator of the PERK kinase function by recruiting BiP to the PERK luminal domain, as discussed above. The cytosolic ERdj6 pool, on the other hand, could regulate PERK kinase function by releasing the translational block during the recovery period.

## 4. Protein Degradation Controlled by ERdj3, ERdj4, and ERdj5

In the ER lumen, protein aggregates activate the UPR, and luminal, misfolded proteins are eventually targeted to the ERAD pathway by the means of which they are retrotranslocated to the cytosol for ubiquitination and subsequent degradation by the proteasome [55,56]. The ERAD pathway consists of several branches with distinct specificities. The central protein of the retrotranslocon channels is Hrd1, which forms a ubiquitin-gated protein-conducting channel [57,58]. Hrd1 is complexed with proteins located luminally—to enable the docking of targets—and a membrane-embedded ubiquitin ligase [59]. All ERAD branches converge in the cytosolic AAA-ATPase (Cdc48 in yeast; p97/VCP in mammals), which assists in the retrotranslocation process and passes the targets to the proteasome [59]. The Sec61 translocon, which is the channel for protein import but, also, for cotranslational degradation (see above), is still under debate for the retrotranslocation of misfolded proteins. Even more so, since mutants of the Sec61 translocon revealed retrotranslocation-specific deficits [60,61]. In any case, it is difficult to differentiate between ERAD-mediated retrotranslocation (via Hrd1-channels) and cotranslational degradation (at the Sec61 channel). We suggest considering the cotranslational degradation at the Sec61 translocon as a specific branch of ERAD, since both (ERAD and cotranslational degradation) are active in stressed cells, converge in the cytosolic AAA-ATPase, result in a decline in the protein levels in the ER lumen and the ER membrane, and in an increase in the ER protein levels upon inhibition of the proteasome. A decline in the protein levels in the ER could be based on a limited protein import or increased protein degradation. The inhibition of the proteasome proved to be a reliable method to distinguish between both mechanisms, since, in the case of ERAD, proteasome inhibition results in increased luminal levels of the respective target protein. In the following, we will decipher the roles of ERdj3, ERdj4, and ERdj5, which are known to assist in the degradation of misfolding proteins along the ERAD pathway (Figure 3b).

The first indications that ERdj3 is involved in the retrotranslocation of substrates was first published in the context of toxin transport from the ER to the cytosol [62]. Vero cells, overexpressing ERdj3 and treated with Shiga toxin, were susceptible to the toxin, while the overexpression of a truncated ERdj3 construct conferred resistance to infected Vero cells. Based on these findings, the authors suggested that truncated ERdj3 exerts a dominant-negative effect on WT ERdj3 and inhibits retrotranslocation from the ER to the cytosol. Since ERdj3 was found in a complex with the toxin and the Sec61 translocon in in vitro experiments, the Sec61 translocon was suggested to function as a retrotranslocon channel for Vero toxin [62]. In addition to ERdj3, ERdj5 was found to be involved in the retrotranslocation of the cholera toxin and SV40 viral particles [63,64,65,66]. These data point to the involvement of ERdj3 and ERdj5 for the retrotranslocation of toxins via the Sec61 channel. Yet, it remained unclear whether toxins and ERAD substrates utilize the same retrotranslocation channel [61]. According to the current model, and as outlined above, a large number of misfolded substrates in the ER lumen are retrotranslocated by the Hrd1 complex, while the retrotranslocation of toxins and cotranslational degradation occur via the Sec61 channel.

### 4.1. Processing of Target Proteins: Futile Folding Versus Degradation of Target Proteins

Misfolded proteins that cannot be refolded can either aggregate and accumulate intra- or extracellularly or are subjected to the ERAD pathway. Degradation via the ERAD can also occur to proteins that carry a mutation but would be functional when folded correctly, as is shown for the mutated chloride channel, resulting in cystic fibrosis disease or mutated β-glucocerebrosidase in Gaucher’s disease [67,68]. Different experimental models have been established to investigate the fate of proteins, especially of those that are known to be associated with human diseases. Some of them are referred to in the following section, such as the amyloid protein with Alzheimer’s disease, α1-antitrypsin with α1-antitrypsin deficiency (AATD), β-glucocerebrosidase (β-GCases) with Gaucher’s disease, the chloride channel and ENaC with cystic fibrosis, or insulin with diabetes (Table 1).

Alzheimer Disease

A prominent experimental system for investigating the degradation pathway of misfolded proteins is the overexpression of the Swedish variant of APP (APPsw), which is known to give rise to aggregation-prone amyloid β (Aβ) peptides, which are deposited intra- and extracellularly. In Alzheimer’s diseased brains, these Aβ peptides derive from the proteolytic cleavage of the amyloid precursor protein (APP) [69]. Overexpression of ERdj3 or ERdj4 resulted in reduced levels of secreted Aβ peptides in APPsw-expressing HEK293 cells. The effect increased when cells were co-transfected with BiP [69]. The reduced amounts of secreted Aβ peptides correlated with the elevated levels of immature APP, suggesting that the maturation of APP is hindered. Furthermore, the overexpression of various ERdj4 mutants revealed that the downregulation of Aβ peptide secretion depends on a functional J-domain [69], indicating that the reduction of Aβ peptide secretion is ATP- and BiP-dependent. 

Since inhibition of the proteasome did not increase the Aβ peptide levels, the ERAD pathway seems not to be involved [69]. It must be assumed that BiP, together with ERdj4 and ERdj3, inhibit the maturation of APP, which subsequently results in reduced amounts of secreted Aβ peptides [69]. Possibly, in the presence of ERdj3/BiP and ERdj4/BiP, ongoing BiP–ATP cycles are run by the cell in a vain attempt to process APP. The fate of the unprocessed APPsw protein has not been investigated. It would be of interest to know whether the inhibition of the proteasome affects the levels of immature APP in the presence of BiP and the co-chaperones ERdj3 and ERdj4, an issue that has not been addressed so far.

α1-Antitrypsin Deficiency

Another protein of clinical relevance is α1-antitrypsin, which is a secreted protease inhibitor. Mutations in the protein can cause α1-antitrypsin deficiency (AATD), resulting in pulmonary emphysema and liver cirrhosis [78]. In human liver cells, it was shown that, within the ER, ERdj3 associates with both the WT α1-antitrypsin (AAT) protein, as well as with the Z variant of α1-antitrypsin (ZAAT), in which a single mutation at position 342 causes the accumulation of homopolymers [70,78,79]. Pulse chase experiments revealed that the downregulation of ERdj3 increases intracellular ZAAT degradation; ERdj3 overexpression, on the other hand, increased the protein levels and aggregation of intracellular ZAAT [70,71], suggesting that ERdj3 delays the degradation of α1-antitrypsin. Similar to the findings in APPsw-overexpressing cells, a chaperoning cycle can be assumed that does not succeed in the proper folding of the mutant protein, thereby delaying its degradation.

Diabetes

Insulin is another target of major clinical relevance. Insulin is synthesized in pancreatic islets and required for lowering the blood sugar level. Dysregulation of insulin maturation, secretion, or degradation can result in diabetes. Co-chaperones either assist in the proper folding of insulin or promote its targeting to the ERAD pathway. In ERdj4 gene-trapped mice (ERdj4^GT/GT^), increased levels of plasma proinsulin, as well as increased proinsulin:insulin ratios, were measured, pointing to a limited maturation of insulin in the absence of ERdj4 [74]. Besides controlling the maturation of insulin, ERdj4 and ERdj5 co-chaperones have been shown to target misfolded proinsulin to the ERAD pathway [67]. Co-immunoprecipitation experiments have further identified the ERAD component Derlin1 as an ERdj4-associated partner, substantiating the involvement of ERdj4 in the ERAD pathway [75]. Additionally, interaction with the cytosolic protein p97/VCP (Cdc48 ATPase in yeast) was shown for ERdj4 and ERdj5 by co-immunoprecipitation experiments [61,67,75]. Since p97/VCP binds to ubiquitinated proteins and assists in the transport of ERAD substrates from the ER to the cytosol, these data further substantiate the involvement of ERdj4 and ERdj5 in the ERAD pathway.

Gaucher’s Disease

Mutations in β-glucocerebrosidase (β-GCases) cause a lysosomal storage disease, Gaucher’s disease [68]. This mutant protein was also shown to be bound by ERdj3. In HeLa cells and human fibroblasts, ERdj3 was shown to bind to WT β-GCase but to bind even better to the mutated β-GCase to target it to the degradation pathway, although it were functional when folded correctly [68]. The knockdown of ERdj3 increased the amount of mutant β-GCase, which is normally barely detectable due to extensive ERAD, indicating that ERdj3 is functionally involved in the degradation pathway that targets destabilized mutant β-GCase [68]. Accordingly, downregulation of ERdj3 resulted in increased folding, transport, and enzyme activity of β-GCase mutants. An analysis of the underlying mechanism revealed that increased amounts of calnexin were attached to the β-GCase mutants in the absence of ERdj3 [68]. Calnexin is an ER resident chaperone that binds to specific sugar chains of glycosylated substrates, thereby promoting the correct folding of these proteins [80]. Likewise, the simultaneous stimulation of calnexin activity using diltiazem and downregulation of ERdj3 increased the amount of folded β-GCase mutants in fibroblasts [68]. Accordingly, ERdj3 seems to prevent calnexin binding to the mutant β-GCase, thereby subjecting mutant proteins to the degradation pathway, while, in the absence of ERdj3, more calnexin can bind to the mutant β-GCase, thereby rescuing it from degradation and enabling its folding [68].

Cystic Fibrosis

Cystic fibrosis is the second-most common pediatric respiratory disease [81]. Cystic fibrosis is a recessive genetic disease that is caused by a mutation in the cystic fibrosis transmembrane conductance regulator gene (CFTR), which encodes a chloride channel expressed in exocrine glands. CFTR also regulates other ion channels, such as the epithelial sodium channel (ENaC), which is required for regulating the osmotic balance and viscosity of the mucus [82]. The fate of both channels seems to be under the control of ERdj4 and, possibly also, of ERdj3. The most common mutation in the CFTR gene is Δ508CFTR, which is rapidly degraded. Inhibition of the ERAD pathway rescues the mutated Δ508CFTR, resulting in a functional chloride channel, which is targeted to the plasma membrane [67]. Similarly, downregulation of *ERdj4* by siRNA increased the levels of functional WT-CFTR and ΔF508CFTR at the plasma membrane. Interestingly, knockout of only one *ERdj4* allele sufficed to rescue the CFTR protein levels. Likewise, overexpression of *ERdj4* increased the levels of ubiquitinated ΔF508CFTR, and even of WT-CFTR, in HEK293 cells. These results suggest that, under physiological conditions, ERdj4 promotes ΔF508-CFTR to ERAD and that downregulation of *ERdj4* decreases the ΔF508-CFTR degradation rates [76]. Recently, by means of a ProtoArray, ERdj3 was also identified as an interaction partner of CFTR [76], indicating that ERdj3 might be involved in the folding and degradation of CFTR. However, this needs to be shown in future experiments. ERdj3—together with its binding partners SDF2L1 and SDF2—has been shown to prevent the protein aggregation of other proteins such as α1-antitrypsin, α1-antitrypsin mutants, denatured GSH S-transferase, or immunoglobin κ light chain [83,84,85].

ERdj3 and ERdj4 also seem to cooperate in controlling the degradation of the ENaC channel. The ENaC is an integral membrane protein that consists of three subunits, 75% of which reside in the ER lumen, whereas 25% reside in the cytoplasm. Experiments on Xenopus oocytes showed a reduced current through the channel due to a reduced surface expression in the presence of ERdj3 or ERdj4. When inhibiting the proteasome with MG-132, ERdj3 had no adverse effect on the current passing through the ENaC channel [77], supporting the notion that both co-chaperones, ERdj3 and ERdj4, deliver the sodium channel ENaC to the ERAD pathway.

Interstitial Lung Disease

Pulmonal surfactant protein-C (SP-C) is required for reducing the surface tension at the air–liquid interface and to prevent the collapse of respiratory tissue. Mutations in the SP-C gene are associated with interstitial lung disease (ILD) [86]. Overexpression of a mutant SP-C variant (SP-C^Δexon4^) in cell culture experiments resulted in the retention of protein aggregates in the ER and activation of the UPR [87]. In HEK293 cells, ERdj4 and ERdj5 were upregulated in response to the expression of mutant SP-C and were shown to bind to the mutant protein [67]. Since inhibition of the proteasome resulted in elevated levels of the surfactant proprotein, misfolded SP-C seems to be an ERAD substrate that is bound and targeted to ERAD by ERdj4 and ERdj5 [67]. No association of ERdj3 and mutant SP-C could be detected in HEK293 cells [67], which suggests that different target sequences are recognized by ERdj3 and ERdj4/ERdj5. In the following section, we decipher the current knowledge on the underlying mechanism that determines the binding of co-chaperones and the fate of the bound target.

### 4.2. Features Determining the Fate of Target Proteins

The data listed above show that BiP and the co-chaperones ERdj3 and ERdj4 have the potential to control both, the maturation and degradation of proteins. It is not clear which parameters determine the fate of the target proteins, resulting in either (futile) chaperoning cycles or in targeting mutant proteins to the ERAD pathway (Table 1). Since targets, especially those bound by ERdj3 or ERdj4, experience different fates, it is of great interest to investigate the underlying mechanisms.

One possibility for directing the fate of target proteins could be the specificity of target sites to which the co-chaperones bind to. In order to characterize the binding sites, a peptide library was screened that was loaded with peptides derived from target proteins of ERdj3, ERdj4, and ERdj5 [88]. This screen revealed that ERdj3 recognizes multiple domains within the two proteins examined, whereas ERdj4 and ERdj5 bind to less, yet aggregation-prone, sequences. Mutations in ERdj4 and ERdj5 that abolished target binding of the co-chaperones resulted in an increase of the half-lives of the examined peptides, indicating that the peptides are degraded upon the binding of ERdj4 and ERdj5. However, insertion of the identified ERdj4- or ERdj5-binding sites into peptides that are not normally bound by both co-chaperones did not accelerate the degradation of the peptides but resulted in increased aggregation. The authors suggested that this might be due to the lack of binding sites for other ERAD components in these peptides [88]. An alternative explanation might be that the insertion of co-chaperone-binding sites alters the equilibrium between aggregation-prone proteins and co-chaperones towards the aggregation-prone peptides, resulting in increased aggregation.

When comparing the interaction of ERdj3 with β-GCase and with mutant α1-antitrypsin protein ZAAT, it becomes obvious that the binding of ERdj3 to the targets alone does not decide upon their future fate. While downregulation of ERdj3 results in increased folding and maturation of β-GCase, downregulation of ERdj3 increases the degradation of ZAAT [68,70]. At first sight, these different outcomes seem contradictory. Yet, both proteins, β-GCase and ZAAT, are glycosylated proteins, and as such, both are subjected to the calnexin/calreticulin cycle to acquire their correct folding and quality control [68,70,79]. The different outcomes—folding versus degradation—are presumably due to this quality control cycle and the duration of the protein’s sojourn, during which ERdj3 might compete with either connexin or calreticulin (Figure 3b). In the absence of Erdj3, more calnexin attaches to β-GCase, and also, more calreticulin is bound to ZAAT [70]. The downregulation of calreticulin results in increased secretion of ZAAT complexed with ERdj3 [79]. It is known that, when calnexin/calreticulin fail to mediate proper folding of glycosylated substrates, the subsequent cleavage of mannosidase residues by ER α1,2-mannosidase I and EDEM1 enables binding of the ER-resident lectins OS-9 and XTP3-B to the misfolded proteins and, further, to the membrane-adaptor protein SEL1L to mediate targeting of the misfolded protein to the retrotranslocon for its degradation by the ERAD pathway [80,89,90]. The reason for the different outcomes must be searched during the competition of the binding of calnexin/calreticulin and ERdj3 to the targets, which determines the outcome of folding versus degradation. These different outcomes could be due to different degrees of misfolding of the two proteins or due to other distinct features, such as that β-GCase is a lysosomal protein while ZAAT is a protein that should normally be secreted.

In conclusion, in both cases, the downregulation of ERdj3 results in a prolonged sojourn of the targets within the calnexin/calreticulin chaperoning cycle, which enables either folding or degradation, depending on the endogenous—still unknown—target features.

Likewise, ERdj4 can affect maturation but also promote degradation of target proteins [67,69,76,77]. The binding of ERdj4 to mutant SP-C was increased by a defective J-domain [67]. However, the ability of ERdj4 to promote protein degradation was shown to require a functional J-domain [67]. As the J-domain of ERdj4 interacts with BiP [91], a functional ERdj4/BiP interaction seems to be crucial for ERdj4-mediated protein degradation. BiP dependency on the degradation of the target proteins was also shown for ERdj3 and ERdj5 [67]. An ERdj5 mutant lacking its J-domain was still able to bind to mutant and WT SP-C. However, when the HPD site (which is the core sequence of the J-domain required for stimulating the ATPase activity of BiP) within ERdj5 was mutated, no effect on the degradation of mutant and WT SP-C could be observed [67]. According to these data, the binding of ERdj5 to ERAD substrates seems to be BiP-independent, while the ERdj5-mediated degradation of substrates only occurs in the presence of BiP [67].

In contrast to ERdj3 and ERdj4, the binding of ERdj5 to mutant targets did not delay their degradation due to a futile chaperoning function. It was shown that ERdj5 promotes the degradation of a set of ERAD substrates (mutant SP-C, mutant insulin, WT insulin, α1-antitrypsin NHK variant, mutant tyrosine kinase, and J-chains) [67,72,92]. This is presumably due to the special structure of ERdj5, which harbors six thioredoxin domains, which confer a strong reductive potential for ERdj5 [93,94]. Due to this reductive strength, ERdj5 can reduce the intra- or intermolecular disulfide bonds, thereby straightening the targets prior to their delivery to the ERAD pathway [72]. This hypothesis is supported by data showing that ERdj5 mutants with defective thioredoxin domains cannot promote the degradation of ERAD substrates [72,92]. In addition, ERdj5 neither promotes the degradation of a cysteine-free mutant of the α1-antitrypsin NHK variant nor of the cysteine-free protein ribophorin [72]. The role of ERdj5 in the ERAD pathway is substantiated by co-immunoprecipitation, as well as by yeast two-hybrid experiments showing the association of ERdj5 with different other ERAD components, such as EDEM1 [72,92,95]. The association of ERdj5 with EDEM1 is not dependent on the reductase activity of ERdj5, as an ERdj5 mutant without a functional thioredoxin domain still binds to EDEM1 [72]. It is rather the C-terminal cluster of ERdj5 that binds to the ERAD targets, as shown for binding to EDEM1 and to the α1-antitrypsin-null variant NHK [92,95]. Two additional components of the ERAD machinery were identified as ERdj5 interaction partners: the ERAD-associated membrane adaptor protein SEL1L [96] and cytosolic AAA-ATPase p97/VCP [67]. Since ERdj5 is located at the luminal side and p97/VCP at the cytosolic side [93,97,98], both proteins presumably bind to the target at either side of the retrotranslocation channel.

Taken together, ERdj3 and ERdj4 can promote the folding or degradation of their target proteins. The exact underlying mechanisms have not yet been solved, but ERdj3 seems to compete with calnexin/calreticulin for binding to the targets and thereby decide on the subsequent fate of the target. ERdj5 predominantly serves as a reductase, releasing a three-dimensional, sterically hindering conformation of the targets. According to the close association of ERdj4 with the retrotranslocon, the “straightened” targets are then presumably handed over to ERdj4 for their subsequent retrotranslocation across the ER membrane.

## 5. Conclusions

Secreted and integral membrane proteins are transported across the ER membrane either co- or post-translationally. Within the ER lumen, proteins acquire their proper three-dimensional conformation by energy-consuming, ER-resident chaperone machinery. The central ER resident chaperone is the Hsp70 protein BiP. The chaperoning activity of BiP is assisted by ER resident co-chaperones. Besides their chaperoning activity, three of them, ERdj1, ERdj2, and ERdj6, have been shown to be engaged in controlling the translation and import of ER target proteins at the Sec61 translocon. This translational and translocational control can be adapted to the prevailing luminal conditions, enabling the arrest of translation and translocation in the case of an increased accumulation of misfolded proteins. In addition to playing decisive roles in protein import, misfolded proteins in the ER lumen are retrotranslocated via ER-associated degradation (ERAD), a pathway controlled and mediated by the co-chaperones ERdj3, ERdj4, and ERdj5. As such, ER resident co-chaperones also coordinate the degradation of proteins to clear the ER from extensive protein loads, processes that play critical roles under normal physiological conditions but, even more, when the UPR is initiated due to changes in the cellular microenvironment.

## Figures and Tables

**Table 1:** Examples of co-chaperone functions in mutant proteins causing metabolic diseases.

Disease	Mutated Protein	Co-Chaperone	Function	Reference
Alzheimer’s disease	Amyloid β-protein	ERdj3 ERdj4	Chaperoning Chaperoning	[69] [69]
α1-Antitrypsin disease (AATD)	α1-Antitrypsin	ERdj3	Chaperoning	[70,71,72,73]
Diabetes	Insulin	ERdj4 ERdj4 ERdj5	Chaperoning ERAD ERAD	[74] [67,75] [67]
Gaucher’s disease	β-Glucocerebroside	ERdj3	ERAD	[68]
Cystic fibrosis	Transmembrane conductive regulator protein			
	Chloride channel	ERdj3 ERdj4	? ERAD	[76] [76]
	ENaC channel	ERdj3 ERdj4	ERAD ERAD	[77] [77]
Interstitial lung disease (ILD)	Surfactant protein-C	ERdj4 ERdj5	ERAD ERAD	[67] [67]

“?”, needs to be confirmed in future studies.

## Data Availability

Not applicable.
